# Therapeutic modalities for iatrogenic late paresthesia in oral tissues innervated by mandibular branch of trigeminal nerve: a systematic review

**DOI:** 10.1186/s40902-024-00438-5

**Published:** 2024-07-15

**Authors:** Elham Keykha, Elahe Tahmasebi, Mahdi Hadilou

**Affiliations:** 1 Research Center for Prevention of Oral and Dental Diseases, Baqiyatallah University of Medical Sciences, Tehran, Iran; 2 Faculty of Dentistry, Research Center for Prevention of Oral and Dental Diseases, Baqiyatallah University of Medical Sciences, Tehran, 1435916471 Iran

**Keywords:** Mandibular nerve injuries, Paresthesia, Systematic review, Trigeminal nerve injuries

## Abstract

**Background:**

The present systematic review intended to evaluate the current evidence on the modalities used for treating iatrogenic late paresthesia in the oral tissues innervated by the mandibular branch of the trigeminal nerve.

**Main text:**

As a common side effect of dental procedures, paresthesia can exert a profound adverse effect on patients’ quality of life. The inferior alveolar nerve (IAN) and lingual nerve (LN) have the highest chance of injury during several dental procedures, including mandibular orthognathic surgeries, implant placement, extraction of the third molar, anesthetic injections, flap elevation, and endodontic treatments. Moreover, several methods have been proposed for treating iatrogenic late paresthesia, including photobiomodulation (PBM), microsurgery, medication, and close observation until achieving spontaneous recovery of sensation. However, no gold standard treatment for iatrogenic paresthesia has been agreed upon up to now. The present study included a comprehensive search of the databases of PubMed, Embase, Scopus, and Web of Science up to December 04, 2023, resulting in a total of 3122 related studies. Then, the titles, abstracts, and full texts of the studies were evaluated. Ultimately, seven controlled randomized trials (RCTs) were included in the final analysis. Also, the risk of bias was assessed using the Joanna Briggs Institute (JBI) critical appraisal checklist. Among all fields, randomization, allocation concealment, and data analysis were found to have the highest chance of bias in the included studies.

**Conclusions:**

In conclusion, PBM, vitamin B12, and corticosteroids could accelerate the recovery of late paresthesia. However, considering the low sample size of the included studies and the high risk of methodological bias, it is recommended to perform further RCTs with robust study designs following Good Clinical Practice (GCP) guidelines to achieve more reliable results.

**Supplementary Information:**

The online version contains supplementary material available at 10.1186/s40902-024-00438-5.

## Background

Iatrogenic or accidental manipulations of the hard and soft tissues of the oral cavity may cause several neurosensory disturbances (NSDs), which may occur as the side effect of mandibular orthognathic surgeries, implant placement, extraction of the third molar, anesthetic injections, flap elevation in anatomically critical areas, endodontic treatments, and traumatic fractures [1–3]. According to reports, the incidence of such disturbances is quite higher than what was thought before. For example, the incidence of NSDs in the manipulations of the lingual nerve (LN) and inferior alveolar nerve (IAN) has been reported to be 0.1–22% and 0.26–8.4%, respectively [4]. The term NSD covers an extensive spectrum of neurosensory impairments, ranging from neuralgia, defined as an abnormal pain sensation in a specific region, to complete numbness. However, paresthesia is the most common form of NSD [5, 6].

Currently, close observation is the most common way to deal with iatrogenic paresthesia, which often heals spontaneously in 1–3 months. However, a substantial number of cases may experience permanent paresthesia. According to a meta-analysis by Padmanabhan et al., the annual incidence of persistent NSD was 18.67% (95% *CI*: 14.54–2.79), which cannot be neglected [2]. On the other hand, NSD can remarkably affect a patient’s quality of life. According to a study by Leung et al., the severity of numbness in the tissues innervated by the IAN and LN has a moderate correlation with the level of quality of life. However, such a correlation was not found in the patients with neuralgia in the same areas [7, 8].

In 1942, Seddon classified paresthesia induced by mechanical injury into tree classes, including neuropraxia, axonotmesis, and neurotmesis, based on histopathological changes of the affected nerve. Neuropraxia is defined as the demyelination of the injured nerve segment while maintaining nerve continuity, while axonotmesis is characterized by the complete interruption of the axon accompanied by the intact connective tissue surrounding the nerve. Finally, the complete severance of the nerve is referred to as neurotmesis. It is obvious that the chance of the recovery of normal sensation is decreased with increased severity of the injury from neuropraxia to neurotmesis [9].

On the other hand, the IAN and LN have the highest possibility of iatrogenic paresthesia among the nerves innervating the oral region [3]. The NSDs in these nerves may affect the sensation of the left or right sides of the mandible. Moreover, the operator-related risk factors of NSDs, including lack of experience and not maintaining a safe distance from the surrounding nerves, can be modified by using imaging modalities providing 3D information in complicated cases. However, there are still several situations in which the nerve injury is inevitable, necessitating the need for treatment [10]. Thus, in addition to close observation, several other therapeutic modalities have been proposed for iatrogenic paresthesia, including medical therapy with vitamin B12 [11], or corticosteroids [12], surgical procedures like microsurgery or nerve grafting [13], photobiomodulation (PBM) [1], electrical stimulation, and local physiotherapy [14]. However, these modalities have achieved controversial results [15–18].

Nevertheless, recent studies have focused on the potential benefits of preemptive application of these treatments as an adjunctive therapy during dental procedures with a high chance of nerve damage. Thus, such studies cannot help in finding the exact role of these therapeutic modalities in treating “late” paresthesia, which is defined as a sensation of paresthesia that lasts at least a week post-operation with no spontaneous recovery. The cutoff used for the duration of late paresthesia has been set to omit the temporary effect of postoperative tissue inflammation, which also can mimic NSD.

Considering the above, as well as the legal consequences of persistent iatrogenic paresthesia for dentists, the present systematic review intended to evaluate the available evidence on the treatments used for paresthesia improvement and sensation recovery in patients with iatrogenic late paresthesia.

The study question is as follows:

“What methods are available to treat iatrogenic late paresthesia in the oral tissues innervated by the mandibular branch of the trigeminal nerve?”

## Material and methods

The present systematic review was designed based on the Cochrane Handbook for Intervention Reviews [19] and Preferred Reporting Items for Systematic Reviews and Meta-Analyses (PRISMA) [20]. Moreover, the study protocol was registered in the International Prospective Register of Systematic Reviews with the registration code of CRD42023447657.

### Eligibility criteria

The present study included all controlled clinical trials specifically evaluating iatrogenic late paresthesia, defined as paresthesia lasting for at least a week following dental procedures, in human subjects. Moreover, studies intentionally starting treatment sooner than a week after a dental procedure were excluded from the present systematic review to eliminate the effect of acute postoperative inflammation. The studies using treatments preemptively or adjunctive to dental procedures and studies without a control group, with retrospective, cohort, case report, case series, and review design, were excluded.

### Databases and search

The databases of PubMed, Embase, Scopus, and Web of Science were searched for related studies on December 04, 2023, by MH. Moreover, the gray literature, including resources, Google Scholar, and ProQuest Dissertation & Theses, was searched to find the theses with related topics. The search was performed using PubMed, MeSH terms, Emtree of Embase, and manual keywords. The references of the included studies were evaluated as well. The search strategies used for each database are available in Supplementary Material 1.

### Study selection and data extraction

The titles and abstracts of the searched studies were screened to fulfill the eligibility criteria. Then, the full texts were evaluated. Screening and data extraction were conducted by EK and MH separately, and the disagreements were resolved by group discussions. In the pilot phase, a data extraction table was designed to summarize the main findings and methodology of each study, including the study type, involved nerve, injury cause and type, the interval between nerve injury and treatment start, study arms, sample size, intervention site and duration, follow-up period, and the criteria used for evaluations.

### Risk-of-bias assessment

The possibility of bias in the studies was evaluated using the Joanna Briggs Institute (JBI) critical appraisal checklist [21] by MH and ET separately, and the disagreements were resolved by discussions with a third author (E. K.). The mentioned checklist is designed exclusively for randomized controlled trials (RCT) and covers all possible fields from which bias may arise, including randomization of sample selection, allocation concealment, blinding on different levels, and statistical analysis. The summary of the risk-of-bias assessments and related graphs was illustrated using the robvis visualization application [22].

## Results

**Fig. 1:**
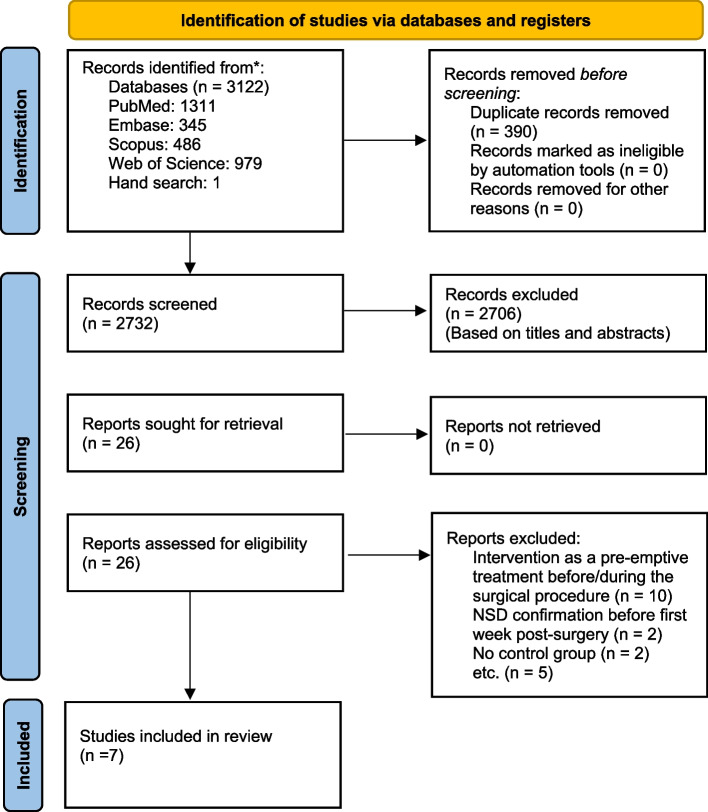
PRISMA flowchart

A total number of 3122 related studies were found by searching, from which 390 were duplicates (Fig. 1). Moreover, 2706 studies were excluded by checking the titles and abstracts, resulting in 26 remaining studies. Finally, 7 out of 26 studies were selected by evaluating their full texts. The causes for the exclusion of the remaining 19 studies are available in Supplementary Material 2.

### Characteristics of included studies

The included studies were conducted in Brazil [14, 17], Denmark [16], Iran [1], Japan [12], the United States [16], Sweden, and Norway [23, 24] and investigated the therapeutic modalities of PBM [1, 14, 16, 23, 24], PBM acupuncture [14, 17], electroacupuncture [17], administration of vit. B [14], and corticosteroids [12]. Only one study had a split-mouth design [17], while others were RCTs with parallel arms. Moreover, one study evaluated both the LN and IAN [16], while others only evaluated the IAN injuries. Also, one study investigated the cases with neurotmesis [1], three included neurapraxia and/or axonotmesis cases [12, 23, 24], and others did not mention the injury type.

The interval between injury time and the beginning of interventions ranged from 7 days to 2 years. Moreover, a study by Salari et al. [1] had the longest follow-up period (12 months). Ultimately, the present systematic review included 222 patients (Table 1). Also, as six out of seven studies had a PBM arm, a table containing PBM parameters and application protocols was designed (Table 2).

**Table 1:** Characteristics of included studies.

**Authors**	**Study type**	**Involved nerve/cause and type of disorder**	**Time delayed after the nerve injury**	**Type of procedure (number of patients)**	**Site and duration of procedure**	**Follow-up period**	**Evaluated criteria**	**Main findings**
Salari et al. (2022) [1]	RCT-parallel	IAN/orthognathic surgery-neurotmesis	At least 6 months	G1: PBM (26) G2: Sham (26)	PBM was applied on 4 points in the buccal site from the oral commissure to the lingual. 12 sessions (2 times/week for 6 weeks)	Before the intervention, immediately after each PBM session, and after 3, 6, 9, and 12 months	Light touch sensations, 2-point discrimination, thermal discrimination, electric pulp test Oral health impact profile (OHIP)-14	PBM improved the light touch sensations and 2-point discrimination. OHIP-14 was significantly higher in intervention group 3 months after PBM. No difference was seen in EPT and thermal discrimination test results between groups.
Oliveira et al. (2021) [14]	RCT-parallel	IAN/third molar or implant surgery-NM	< 90 days	G1: PBM (20)	The IAN innervated tissues. 20 sessions (2 times/week for 10 weeks)	Before the intervention, immediately after the treatment, 5 and 10 weeks	General perception of paresthesia (VAS), thermal discrimination, vibratory mechanical perception threshold, 2-point discrimination threshold, pain perception threshold (VAS), tactile perception threshold	All treatments improved the neurosensory recovery. PBM and medication were superior than PBM acupuncture. There was no difference between PBM and medication group.
				G2: PBM acupuncture (20)	Applied on 6 points: ST 4, M-HN-18, CV 24, ST 5, ST 6, and A1. 20 sessions (2 times/week for 10 weeks)	Before the intervention, immediately after the treatment, 5 and 10 weeks		
				G3: Medication (vitamin B12, cytidine monophosphate, uridine trisodium triphosphate, pyrimidine ribonucleotides) (20)	4 weeks of treatment (1 capsule, every 8 h, 30 days)	Before the intervention, immediately after the treatment, 4 and 8 weeks		
Miloro and Criddle (2018) [16]	RCT-parallel	IAN and LN/third molar surgery, dental implant placement, or local anesthetic injection-NM	> 90 days	G1: PBM (15) G2: Sham (13)	IAN: The IAN path on the affected side (intra-/extraoral) was irradiated. LN: Lingual side of the first and second mandibular molars and lateral of the tongue. 20 sessions in 3 months	Before the intervention and at each treatment visit, 1 week and 3 months after the intervention	Subjective VAS assessment, Brushstroke directional discrimination, 2-point discrimination, contact detection, thermal discrimination	No difference was observed between two groups.
Oliveira et al. (2017) [17]	RCT-split mouth	IAN/orthognathic surgery and genioplasty-NM	7 days	G1: Electroacupuncture (15) G1: Control (15)	Applied on ST 4, M-HN 18, CV 24, ST 5, ST 6, and A1 acupuncture points. 30 sessions (2 times/week for 4 months)	Before the beginning of the treatments and before each clinical session (immediately before the therapeutic intervention).	2-point discrimination test, electric pulp test	There were no statistically significant differences among the groups for the 2-point discrimination test on the buccal mucosa region and for the pulp test on all evaluated regions. The tactile sense improved significantly in electroacupuncture arm.
				G2: PBM acupuncture (15) G2: control (15)	Twice per week on same points as electrical stimulation 30 sessions (2 times/week for 4 months)			
Seo et al. (2004) [12]	RCT-parallel	IAN/orthognathic surgery-neurapraxia or axonotmesis	7 days	G1: Medication (prednisolone) 1-week post-surgery (6) G2: Medication (prednisolone) 3 weeks post-surgery (6) G3: Medication (prednisolone) 6 weeks post-surgery (8) G4: No steroid treatment (6)	Prednisolone was administered for 2 weeks orally to the patients at 30 mg for 7 days, 15 mg for 4 days, and an additional 5 mg for 3 days.	Before the treatment, 1 month after the treatment ended.	Mechanical-touch threshold, heat and cold sensations	Corticosteroids significantly improved mechanical-touch threshold, heat and cold sensation recovery.
Khullar et al. (1996) (a) [23]	RCT-parallel	IAN/orthognathic surgery-neurapraxia or axonotmesis	2 years	G1: PBM (8) G2: Sham (5)	PBM was irradiated along the sites innervated by IAN 20 sessions in 36 to 69 days (mean: 31 days)	Before the treatment, at the end of the treatment	Paresthesia area, thermoceptor neurosensory deficit, mechanoceptor neurosensory deficit, subjective neurosensory deficit (VAS)	PBM improved both subjective and objective mechanoreceptor perception in late IAN paresthesia. The paresthesia area decreased significantly in PBM group. No difference was observed in thermoceptor neurosensory improvement between groups.
Khullar et al. (1996) (b) [24]	RCT-parallel	IAN/orthognathic surgery, third molar surgery, or mandibular fracture-neurapraxia or axonotmesis	At least 6 months	G1: PBM (6) G2: Sham (7)	PBM was irradiated along the sites innervated by IAN 20 sessions in 36 to 69 days (mean: 31 days)	Before the treatment, at the end of the treatment	Thermoceptor neurosensory deficit, mechanoceptor neurosensory deficit, subjective neurosensory deficit (VAS)	PBM improved both subjective and objective mechanoreceptor perception in late IAN paresthesia. No difference was observed in thermoceptor neurosensory improvement between groups.

*NM* Not mentioned, *IAN* Inferior alveolar nerve, *LN* Lingual nerve, *PBM* Photobiomodulation, *VAS* Visual analogue scale

**Table 2:** PBM parameters and application protocols

**Authors**	**Type of laser**	**Wavelength (nm)**	**Mode**	**Output power (mW)**	**Exposure time per point/session**	**Beam diameter (µm-mm)/probe spot size ** $({{\varvec{c}}{\varvec{m}}}^{2})$	**Energy density (** $^{\mathbf{J}}\!\left/ \!_{{{\varvec{c}}{\varvec{m}}}^{2}}\right.$**)**	**Distance to irradiation site**
Salari et al. (2022) [1]	GaAlAs diode laser (THOR LX2, manufactured by Thor international Ltd., Amersham Bucks, UK)	810	Continuous	200	30 s per point (4–6 points)	0.364	12–14	In contact
Oliveira et al. (2021) [14]	InGaAIP diode laser (Therapy XT, DMC Equipamentos, São Carlos, SP, Brazil)	808	Continuous	100	40 s per point (6 points in PBM acupuncture, 26 points in PBM)	0.028	142.8	In contact
Miloro and Criddle (2018) [16]	Diode laser (PHOTON*PLUS System; Rønvig A/S, Daugaard, Denmark)	830	Continuous	360–400	8 points	0.15	NM	In contact
Oliveira et al. (2017) [17]	A low-power laser (780 nm, Laser Hand, MMOptics, São Carlos, SP, Brazil)	780	Continuous	70	6 s per point (6 points)	0.04	10	In contact
Khullar et al. (1996) (a) [23]	GaAlAs diode laser (Rtnvig A/S, Vejle, Denmark)	820	Continuous	70	85 s per point (4 points)	0.13	48	In contact
Khullar et al. (1996) (b) [24]	GaAlAs diode laser (Rtnvig A/S, Vejle, Denmark)	820	Continuous	70	90 s per point (4 points)	0.13	48	In contact

*NM* Not mentioned, *nm* Nanometer, *mW* Milliwatt, *J* Joule, *cm* Centimeter, *s* Second

### Risk-of-bias assessment

**Fig. 2:**
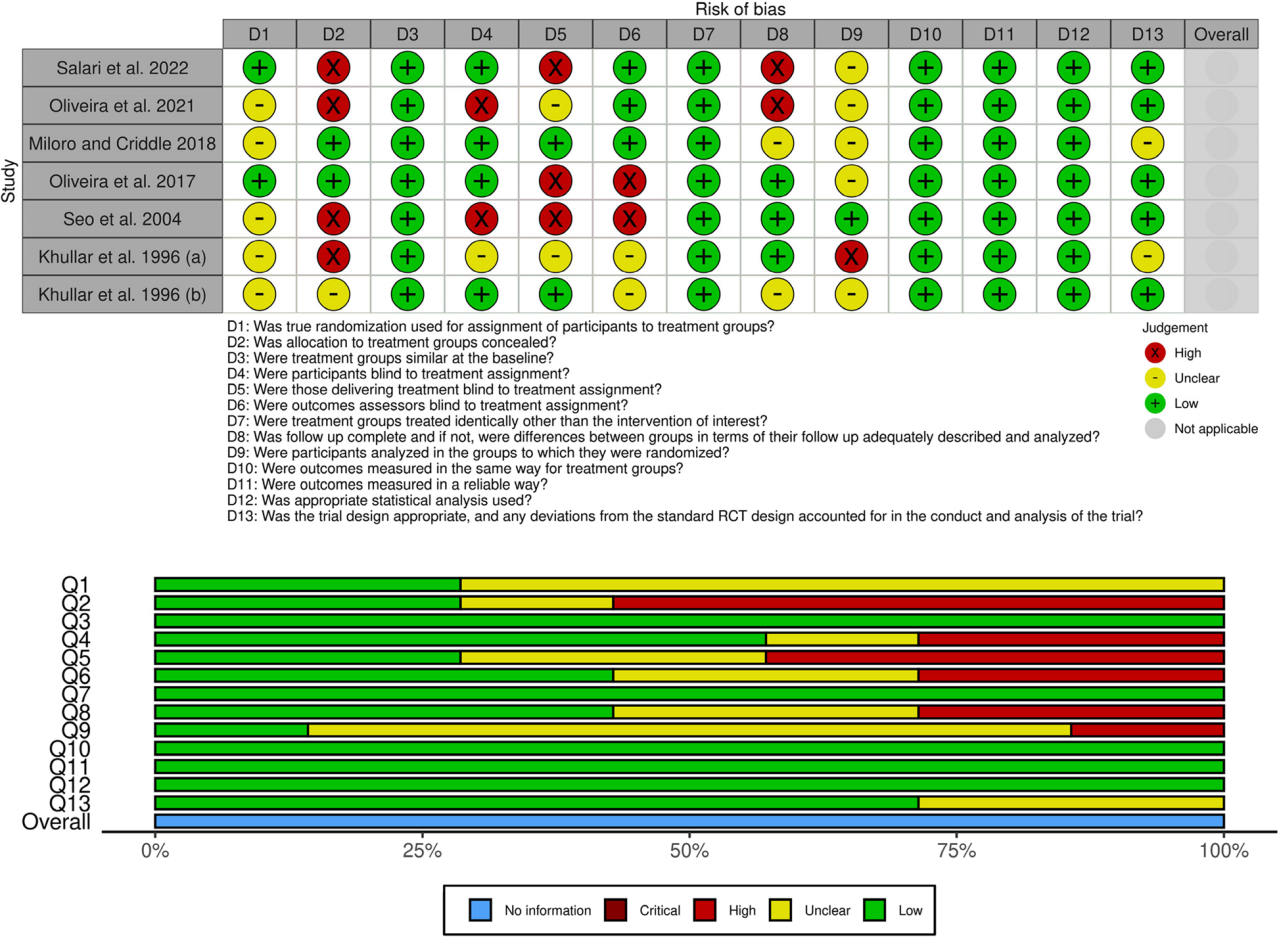
Risk-of-bias assessment summary

The summary of the risk of bias in the included studies is presented in Fig. 2. Among all fields, three had the highest rate of bias: randomization (Q1), allocation concealment (Q2), and mentioning the number of patients leaving the study and how this change was included in the statistical analyses (Q9). Also, there were some concerns regarding the participant blinding in some studies, which could affect the validity of related results.

## Discussion

The present study has evaluated the therapeutic modalities available for late paresthesia caused by dental procedures or accidents. Currently, studies have mostly focused on preemptive treatments of nerve damage or the treatments adjunctive to dental procedures. Thus, finding a suitable treatment for late paresthesia has been overlooked, and a gold standard treatment has not been agreed upon yet. Moreover, it is worth noting that acute inflammatory mediators, tissue remodeling, and increased hydrostatic pressure of the manipulated tissue may disturb nerve function in the first week following a dental procedure, leading to oral paresthesia. If this condition remains over a week and does not heal spontaneously, it is called late paresthesia.

On the other hand, it was not possible to perform a comprehensive meta-analysis for such a topic, considering the limited number of related studies and their extensive methodologic heterogeneity. For example, the two-point discrimination test was conducted using a caliper [1], two-pronged dry compass [14], or Mackinnon-Dellon Disk-Criminator [16] in the included studies. Moreover, the initial distance between the tips, their sharpness, and even the pressure applied to the skin were not the same in different studies. Such heterogeneity could affect the obtained results.

The treatments proposed for sensation recovery can act through different mechanisms. For example, vitamin B12 (hydroxocobalamin) is one of the treatments recommended for improving nerve damage, considering its role in cellular proliferation and growth, synthesis of nucleoproteins and myelin, repairing peripheral nerves, and improving neural impulse conduction and myelination [25–27]. Moreover, one of the evaluated studies in the present systematic review compared the effects of PBM and a combination of vit. B12 and pyrimidine ribonucleotides, including cytidine monophosphate and uridine triphosphate trisodium, which were administered every 8 h for 30 days, on late paresthesia, reporting the beneficial effect of vit B12 on sensation improvement [14]. In a 4-week follow-up, PBM was shown to be significantly more beneficial compared to vitamin B12. However, the group receiving vitamin B12 showed better results compared to the PBM group in a limited number of outcomes such as pain perception. Despite these findings, medical therapy may be preferred by the patients because it is more cost-effective than PBM and eliminates the need for an operator and several visits to a healthcare facility.

As the other group of medications used in the related studies, corticosteroids are commonly prescribed following craniomaxillofacial surgeries for alleviating postoperative pain and edema. However, the studies evaluating their therapeutic effects on paresthesia have reported controversial results [28]. A controlled study by Seo et al. evaluated the effect of prednisolone administration (the related protocol is presented in Table 1), and the time of treatment starts on alleviating late paresthesia, reporting that prednisolone administration 3–6-week post-operation might accelerate the sensation recovery. Moreover, other corticosteroids were used during oral surgeries but showed no significant benefit [29]. Thus, there is a need for further studies with larger sample sizes and robust designs to evaluate the effect of corticosteroids on late paresthesia. However, the adverse effects of long-term corticosteroid use, including adrenal insufficiency, osteoporosis, and gastrointestinal, hepatic, and ophthalmologic impacts, should not be neglected [30].

Among the four studies evaluating the therapeutic effect of PBM, four studies showed its significant benefit compared to the control group even in the case of neurotmesis [1]. These findings are compatible with a meta-analysis by Firoozi et al. [31] that investigated the effect of preemptive PBM used during sagittal split ramus osteotomy on the recovery of postoperative NSDs. The meta-analysis included studies using PBM for 6–14 sessions and reported that 8–10 PBM sessions could accelerate the NSD recovery. However, the present systematic review found that more sessions of PBM, about 12–30 sessions, are needed for sensation improvement in late paresthesia.

Compared to surgical and medical therapies, PBM is a noninvasive therapeutic modality with no side effects. However, its application needs an operator. Moreover, the related treatment course is too time-consuming and needs several sessions. According to studies, PBM can alter the electrical activity and morphology of healthy and injured neurons in rats [16]. Also, it has shown beneficial effects in muscle and nerve repair and wound healing by accelerating cellular proliferation, improving local microcirculation, and reducing the inflammatory mediator release in the arachidonic acid pathway, which helps in stabilizing the neuronal membrane and reducing the transmission of pain sensation [1, 32].

Preemptive medical therapy to reduce the occurrence of postoperative paresthesia does not seem to be reasonable for several reasons. Firstly, the prescription of high-dose corticosteroids can result in adverse side effects. Additionally, multiple therapeutic sessions and the associated costs of PBM do not justify its use as a preemptive therapeutic approach. Therefore, it is advisable to wait at least 1 week following surgery to allow for the reduction of postoperative inflammation, confirm the presence of paresthesia, and then commence medical interventions [12, 33]. Furthermore, relying on close observation for spontaneous healing, a common practice, may not be ideal. Each month that passes without intervention reduces the likelihood of recovery by 5.8% [34]. Thus, it is recommended to initiate the aforementioned nonaggressive treatments, either alone or in combination, following the confirmed diagnosis of paresthesia 1-week post-surgery. This approach should take into account the patient’s underlying conditions, the affordability of the treatment, and the time the patient can dedicate to such therapies.

There is limited evidence evaluating surgical treatments for nerve injuries involving both the IAN and lingual nerves. The aggressiveness of these interventions correlates with the severity of the nerve damage. In cases of nerve damage suspected to be due to compression, decompression through internal and external neurolysis is performed by removing or releasing the surrounding tissues to alleviate the pressure. When the two ends of the nerve can reach each other without tension, direct neurorrhaphy is employed; otherwise, the sleeves technique is used. In instances of severely disrupted nerves, an autologous nerve graft, harvested from the greater auricular or medial antebrachial nerves, is utilized. This method is considered the gold standard despite its disadvantages, such as the requirement for a second surgical site [35, 36]. Surgical intervention is generally recommended if sensory recovery has not occurred within 3 to 9 months despite the application of the aforementioned noninvasive modalities [37]. More robust RCTs are needed in the field of surgical nerve damage treatment. These studies should consider the development of next-generation conduits enriched with stem cells, Schwann cells, and extracellular matrix proteins, which enable the controlled delivery of neurotrophic factors for guided nerve regrowth [36].

Finally, the included studies had a considerable amount of bias in several fields, including randomization, allocation concealment, blinding, and data analysis in case of study withdrawal by some patients (Fig. 2). Most studies only mentioned that they used randomization without giving further information on their way of randomization. However, based on the checklists of risk of bias assessment and Good Clinical Practice (GCP) guidelines, such information is not enough [38], and the randomization type (e.g., simple, block, stratified, and others) and related method or tool should be discussed in details. The method of allocation concealment (e.g., using opaque envelopes) should be explained in detail as well. Moreover, most studies only mentioned that they blinded the participants, researchers, or data analysis level, lacked a robust blinding setup, and did not give any further information on the method of maintaining the blinding chain during the study, its potential compromise, and related reasons. Also, the last field with the highest bias was the method of data analysis in case of study withdrawal by some patients. Ultimately, it is recommended to perform future studies addressing the mentioned shortcomings while following the GCP guidelines to achieve valid and reliable results.

## Conclusions

In conclusion, PBM, vitamin B12, and corticosteroids could significantly accelerate the recovery of late paresthesia. However, considering the low sample size of the included studies and the high risk of methodological bias, it is recommended to perform further RCTs with robust study designs to achieve more reliable results.

## Supplementary Information

Supplementary Material 1. Search strategies used in the online databases.

Supplementary Material 2. List of excluded articles with reasons.

## Data Availability

The datasets used and/or analyzed during the current study are available from the corresponding author on reasonable request.

## Abbreviations

IAN: Inferior alveolar nerve
LN: Lingual nerve
PBM: Photobiomodulation
RCT: Randomized controlled trial
NSD: Neurosensory disturbance
