# Supplementary material for: Therapeutic modalities for iatrogenic late paresthesia in oral tissues innervated by mandibular branch of trigeminal nerve: a systematic review
Source: Maxillofac Plast Reconstr Surg. 2024 Jul 15;46(1):25. doi: 10.1186/s40902-024-00438-5 (PMC11637152; doi:10.1186/s40902-024-00438-5)
Supplement: Supplementary file 1 — Supplementary Material 1. Search strategies used in the online databases. [file 40902_2024_438_MOESM1_ESM.docx]

**PubMed**

((((((((("Mouth"[Mesh]) OR ('oral cavity'[Text Word])) OR (oral[Text Word])) OR (dentistry[Text Word])) OR (dental[Text Word])) OR (tooth[Text Word])) OR (teeth[Text Word])) OR (Mouth[Text Word])) AND (((((((((((((((((((((((((((((((("Trigeminal Nerve Injuries"[Mesh]) OR ("Lingual Nerve Injuries"[Mesh])) OR (Lingual Neuropathy, Traumatic[Title/Abstract])) OR (Lingual Nerve Contusion*[Title/Abstract])) OR (Lingual Nerve Transection*[Title/Abstract])) OR ("Mandibular Nerve Injuries"[Mesh])) OR (Mandibular Nerve Injur*[Title/Abstract])) OR (Inferior Alveolar Nerve Injur*[Title/Abstract])) OR (Mental Nerve Injur*[Title/Abstract])) OR (nerve damage[Title/Abstract])) OR (nerve injur*[Title/Abstract])) OR (Lingual Nerve injur*[Title/Abstract])) OR (paraesthesia[Title/Abstract])) OR ("Paresthesia"[Mesh])) OR (Paresthesia[Title/Abstract])) OR (Paresthesias[Title/Abstract])) OR ("Hypesthesia"[Mesh])) OR (Hypoesthesia*[Title/Abstract])) OR (impaired sensation*[Title/Abstract])) OR ('Reduced Sensation'[Title/Abstract])) OR ('neurosensory alteration' [Title/Abstract])) OR (neuropraxia[Title/Abstract])) OR (Neurotmesis[Title/Abstract])) OR (Neurotmeses[Title/Abstract])) OR (Axonotmesis[Title/Abstract])) OR (Axonotmeses[Title/Abstract])) OR (neuropraxy[Title/Abstract])) OR (neurosensory disturbance[Title/Abstract])) OR (neurosensory deficit[Title/Abstract])) OR (reduced sensation*[Title/Abstract])) OR (nerve trauma[Title/Abstract])) OR (nerve contusion[Title/Abstract])))

**Embase**

('nerve trauma':ti,ab OR 'neurosensory deficit':ti,ab OR 'neurosensory disturbance':ti,ab OR neuropraxy:ti,ab OR axonotmeses:ti,ab OR axonotmesis:ti,ab OR neurotmeses:ti,ab OR neurotmesis:ti,ab OR neuropraxia:ti,ab OR 'neurosensory alteration':ti,ab OR 'neurosensory alterations':ti,ab OR 'reduced sensation':ti,ab OR 'impaired sensation':ti,ab OR 'hypoesthesia':ti,ab OR 'hypesthesia':ti,ab OR 'paresthesias':ti,ab OR 'paresthesia':ti,ab OR 'paraesthesia':ti,ab OR 'lingual nerve injury':ti,ab OR 'lingual nerve injuries':ti,ab OR 'nerve injury':ti,ab OR 'nerve injuries':ti,ab OR 'nerve damage':ti,ab OR 'mental nerve injury':ti,ab OR 'mental nerve injuries':ti,ab OR 'inferior alveolar nerve injury':ti,ab OR 'inferior alveolar nerve injuries':ti,ab OR 'mandibular nerve injury':ti,ab OR 'mandibular nerve injuries':ti,ab OR 'lingual nerve transection':ti,ab OR 'lingual nerve contusion':ti,ab OR 'nerve contusion':ti,ab OR 'trigeminal nerve injuries':ti,ab OR 'trigeminal nerve injury':ti,ab OR 'neurotmesis'/exp OR 'neurotmesis' OR 'nerve injury'/exp OR 'nerve injury' OR 'neuropraxia'/exp OR 'neuropraxia' OR 'paresthesia'/exp OR 'paresthesia' OR 'hypesthesia'/exp OR 'hypesthesia' OR 'tongue hypesthesia'/exp OR 'tongue hypesthesia' OR 'neurosensory disturbance'/exp OR 'neurosensory disturbance' OR 'axonotmesis'/exp OR 'axonotmesis') AND ('mouth'/exp OR 'mouth' OR 'oral cavity':ti,ab OR 'mouth cavity':ti,ab OR 'dentistry':ti,ab) AND ([randomized controlled trial]/lim OR 'controlled clinical trial'/de) AND [article]/lim

**Scopus**

( TITLE-ABS-KEY ( "nerve trauma" OR "neurosensory deficit" OR "neurosensory disturbance" OR "neuropraxy" OR "axonotmeses" OR "axonotmesis" OR "neurotmeses" OR "neurotmesis" OR "neuropraxia" OR "neurosensory alteration" OR "neurosensory alterations" OR "reduced sensation" OR "impaired sensation" OR "hypoesthesia" OR "hypesthesia" OR "paresthesias" OR "paresthesia" OR "paraesthesia" OR "lingual nerve injury" OR "lingual nerve injuries" OR "nerve injury" OR "nerve injuries" OR "nerve damage" OR "mental nerve injury" OR "mental nerve injuries" OR "inferior alveolar nerve injur*" OR "mandibular nerve injur*" OR "lingual nerve transection" OR "lingual nerve contusion" OR "nerve contusion" OR "trigeminal nerve injury" OR "trigeminal nerve injuries") ) AND ( TITLE-ABS-KEY ( "mouth" OR "oral cavity" OR "dentistry" OR "oral") ) AND ( TITLE-ABS-KEY ( "clinical trial" OR "clinical study" OR "controlled trial" OR "randomized controlled trial" OR "randomized controlled trial")) AND ( LIMIT-TO ( SUBJAREA,"DENT" ) )

**Web of Science**

1: TI=( 'nerve trauma' OR 'neurosensory deficit' OR 'neurosensory disturbance' OR 'neuropraxy' OR 'axonotmeses' OR 'axonotmesis' OR 'neurotmeses' OR 'neurotmesis' OR 'neuropraxia' OR 'neurosensory alteration' OR 'neurosensory alterations' OR 'reduced sensation' OR 'impaired sensation' OR 'hypoesthesia' OR 'hypesthesia' OR 'paresthesias' OR 'paresthesia' OR 'paraesthesia' OR 'lingual nerve injury' OR 'lingual nerve injuries' OR 'nerve injury' OR 'nerve injuries' OR 'nerve damage' OR 'mental nerve injury' OR 'mental nerve injuries' OR 'inferior alveolar nerve injury' OR 'inferior alveolar nerve injuries' OR 'mandibular nerve injury' OR 'mandibular nerve injuries' OR 'lingual nerve transection' OR 'lingual nerve contusion' OR 'nerve contusion' OR 'trigeminal nerve injury' OR 'trigeminal nerve injuries' )

2: AB=( 'nerve trauma' OR 'neurosensory deficit' OR 'neurosensory disturbance' OR 'neuropraxy' OR 'axonotmeses' OR 'axonotmesis' OR 'neurotmeses' OR 'neurotmesis' OR 'neuropraxia' OR 'neurosensory alteration' OR 'neurosensory alterations' OR 'reduced sensation' OR 'impaired sensation' OR 'hypoesthesia' OR 'hypesthesia' OR 'paresthesias' OR 'paresthesia' OR 'paraesthesia' OR 'lingual nerve injury' OR 'lingual nerve injuries' OR 'nerve injury' OR 'nerve injuries' OR 'nerve damage' OR 'mental nerve injury' OR 'mental nerve injuries' OR 'inferior alveolar nerve injury' OR 'inferior alveolar nerve injuries' OR 'mandibular nerve injury' OR 'mandibular nerve injuries' OR 'lingual nerve transection' OR 'lingual nerve contusion' OR 'nerve contusion' OR 'trigeminal nerve injury' OR 'trigeminal nerve injuries' )

3: #2 OR #1

4: TI=( 'mouth' OR 'oral cavity' OR 'dentistry' OR 'oral' )

5: AB=( 'mouth' OR 'oral cavity' OR 'dentistry' OR 'oral' )

6: #5 OR #4

7: #3 AND #6

8: TS=( 'clinical trial' OR 'clinical study' OR 'controlled trial' OR 'randomized controlled trial' OR ' OR 'randomized controlled study' )

9: #7 AND #8
