# Supplementary material for: Therapeutic modalities for iatrogenic late paresthesia in oral tissues innervated by mandibular branch of trigeminal nerve: a systematic review
Source: Maxillofac Plast Reconstr Surg. 2024 Jul 15;46(1):25. doi: 10.1186/s40902-024-00438-5 (PMC11637152; doi:10.1186/s40902-024-00438-5)
Supplement: Supplementary file 2 — Supplementary Material 2. List of excluded articles with reasons. [file 40902_2024_438_MOESM2_ESM.docx]

| **Supplementary file 2. List of excluded articles with reasons.** | **Reason for exclusion** |
| --- | --- |
| Low-level laser effect in patients with neurosensory impairment of mandibular nerve after sagittal split ramus osteotomy. Randomized clinical trial, controlled by placebo | NSD confirmation before the first week post-surgery |
| Laser Biophotomodulation in Patients with Neurosensory Disturbance of the Inferior Alveolar Nerve After Sagittal Split Ramus Osteotomy: A 2-Year Follow-Up Study | NSD confirmation before the first week post-surgery |
| Does leucocyte- and platelet-rich fibrin enhance neurosensory recovery after genioplasty? A double-blind, split-mouth, randomised clinical trial | Intervention as a pre-emptive treatment before/during the surgical procedure |
| Effect of concentrated growth factor on lower lip hypoesthesia after osseous genioplasty: a prospective, split-mouth, double-blind randomized controlled trial | Intervention as a pre-emptive treatment before/during the surgical procedure |
| Comparison of functional recovery of infraorbital nerve paresthesia following open and closed reduction of zygomaticomaxillary complex fractures | Intervention as a pre-emptive treatment before/during the surgical procedure |
| Evaluation of recovery period in lower lip hypoesthesia after bilateral sagittal split osteotomy using trigeminal somatosensory evoked potential | Retrospective study |
| Effect of photobiomodulation on recovery from neurosensory disturbances after sagittal split ramus osteotomy: a triple-blind randomised controlled trial | Intervention as a pre-emptive treatment before/during the surgical procedure |
| Can platelet-rich fibrin accelerate neurosensory recovery following sagittal split osteotomy? A double-blind, split-mouth, randomized clinical trial | Intervention as a pre-emptive treatment before/during the surgical procedure |
| Does Dexamethasone Facilitate Neurosensory Function Regeneration After Zygomatic Fracture? A Randomized Controlled Trial | Intervention as a pre-emptive treatment before/during the surgical procedure |
| Is Low-Level Laser Therapy Effective for Treatment of Neurosensory Deficits Arising From Sagittal Split Ramus Osteotomy? | Intervention as a pre-emptive treatment before/during the surgical procedure |
| Effects of Superpulsed, Low-Level Laser Therapy on Neurosensory Recovery of the Inferior Alveolar Nerve | No control group |
| Outcome Following Lingual Nerve Repair With Vein Graft Cuff: A Preliminary Report | No control group |
| The effect of a platelet-rich fibrin conduit on neurosensory recovery following inferior alveolar nerve lateralization: a preliminary clinical study | Retrospective study |
| Effect of infrared laser in the prevention and treatment of paresthesia in orthognathic surgery | Intervention as a pre-emptive treatment before/during the surgical procedure |
| The comparative estimation of the efficacy of fluctuorization and ipidacrine fluctuophoresis included in the combined treatment of the patients with lesions in the inferior alveolar nerve | Not in English (in Russian) |
| Microsurgical repair of the peripheral trigeminal nerve after mandibular sagittal split ramus osteotomy | Retrospective study |
| Neurosensory recovery after trauma to the orbital floor: aprospective trial with dexamethasone | Intervention as a pre-emptive treatment before/during the surgical procedure |
| Evaluation of the Treatment Modalities for Neurosensory Disturbances of the Inferior Alveolar Nerve Following Retromolar Bone Harvesting for Bone Augmentation | No specific determination of the neurosensory disturbance type (paresthesia, dysesthesia or neuralgia) |
| Lower-level laser therapy improves neurosensory disorders resulting from bilateral mandibular sagittal split osteotomy: A randomized crossover clinical trial | Intervention as a pre-emptive treatment before/during the surgical procedure |
